# Objective Church Environment Audits and Attendee Perceptions of Healthy Eating and Physical Activity Supports within the Church Setting

**DOI:** 10.3390/ijerph17103598

**Published:** 2020-05-20

**Authors:** Marilyn E. Wende, Andrew T. Kaczynski, John A. Bernhart, Caroline G. Dunn, Sara Wilcox

**Affiliations:** 1 Department of Health Promotion, Education, and Behavior, Arnold School of Public Health, University of South Carolina, Columbia, SC 29208, USA; Mwende@email.sc.edu (M.E.W.); cdunn@hsph.harvard.edu (C.G.D.); 2 Prevention Research Center, Arnold School of Public Health, University of South Carolina, Columbia, SC 29208, USA; WILCOXS@mailbox.sc.edu; 3 Department of Exercise Science, Arnold School of Public Health, University of South Carolina, Columbia, SC 29208, USA; bernhaj@email.sc.edu

**Keywords:** church environment, faith-based setting, health behavior, perceived environment, self-efficacy

## Abstract

Interventions in faith-based settings are increasingly popular, due to their effectiveness for improving attendee health outcomes and behaviors. Little past research has examined the important role of the church environment in individual-level outcomes using objective environmental audits. This study examined associations between the objectively measured physical church environment and attendees’ perceptions of physical activity (PA) and healthy eating (HE) supports within the church environment, self-efficacy for PA and HE, and self-reported PA and HE behaviors. Data were collected via church audits and church attendee surveys in 54 churches in a rural, medically underserved county in South Carolina. Multi-level regression was used to analyze associations between the church environment and outcomes. Physical elements of churches were positively related to attendees’ perceptions of church environment supports for PA (B = 0.03, 95% CI = 0.01, 0.05) and HE (B = 0.05, 95% CI = 0.01, 0.09) and there was a significant interaction between perceptions of HE supports and HE church environment. Self-efficacy and behaviors for PA and HE did not show an association with the church environment. Future research should establish a temporal relationship between the church environment and these important constructs for improving health. Future faith-based interventions should apply infrastructure changes to the church environment to influence important mediating constructs to health behavior.

## 1. Introduction

Physical activity (PA) and healthy eating (HE) are important for maintaining energy balance and reducing risk for obesity [1,2]. PA and HE can also reduce risk for numerous chronic diseases [3,4] and overall mortality [5]. Despite knowing these benefits, a large proportion of United States (U.S.) residents do not meet PA guidelines or consume enough fruits and vegetables [6]. Interventions involving faith-based organizations offer opportunities to increase levels of PA and HE since they facilitate reach within underrepresented groups. In the U.S., approximately 75% of individuals report religious affiliation and 36% report weekly church attendance [7]. Affiliation is highest among older adults, African Americans, and Southeastern residents [7].

Faith-based organizations, such as churches and other groups united on the basis of religious belief, promote PA and HE through social interaction and education and through existing physical structures, such as food resources and activity spaces [8,9,10]. While social and physical environments are the focus of current health promotion research [11], their application within faith-based organizations is understudied [8]. Consistent with social cognitive theory, individual characteristics, such as self-efficacy and perceptions of the environment, may interact with the environmental resources to support healthy behaviors [12,13,14]. Self-efficacy is defined as confidence that one has an influence over his or her behaviors [12], and has not been studied in relation to church environment in the past. In addition, since church environments vary greatly with respect to available health-promoting resources [8,15], perceptions of social and physical supports within them may be important to improve health behaviors. Past research even shows that perceptions of health promotion within the church among attendees and church leadership are related [16], and may also translate to improved health behaviors [17]. 

Some research has looked at the impact of environmental interventions on church member perceptions of the church environment, self-efficacy, and health behaviors, but measurement of these environments were often subjective [18,19,20]. For example, one study observed the role of perceived church environment on health behaviors and showed that providing messaging in sermons and church bulletins, HE programs, and healthy foods was associated with increased HE but not PA [18,19]. This research did not measure any elements of the PA environment in relation to PA behavior, which might explain the non-significant results. Another study looked at differences in perceived environmental resources for PA and HE among black and white church attendees, but responses were qualitative and did not establish a connection between resources available and individual outcomes [20]. Further research is needed to determine whether objective environment audits of PA and HE resources within the church are related to church member perceptions of that environment. Moreover, very little research exists examining whether objective measures of the church environment might also impact PA and HE behaviors, and self-efficacy for those behaviors (which is often a precursor for the behavior itself) [12]. 

Given the aforementioned considerations, the overall purpose of this study was to examine associations between the objectively measured, physical church environment and attendees’ perceptions of the environment and health behaviors. We explored four related research questions: (1) Is the church physical environment positively associated with attendees’ perceptions of the PA and HE supports within the church environment? (2) Is the church physical environment positively associated with attendees’ self-efficacy for PA and HE? (3) Is the church physical environment positively associated with attendees’ self-reported PA participation and fruit and vegetable intake? and (4) Does weekly church attendance modify the relationship between church environments and perceptions of church environment supports, self-efficacy, and behaviors related to PA and HE?

## 2. Materials and Methods

### 2.1. Study Setting and Data Collection

This observational study occurred in a rural, medically underserved county in South Carolina. The county has approximately 23,956 residents [21], with higher levels of poverty, lower levels of education [22], and more negative health outcomes compared to state-wide trends [23]. Data were collected during Phase 1 of a two-phase dissemination and implementation trial of a faith-based intervention to promote PA and HE, which is described in detail elsewhere [9]. Of the 132 active churches in the study county, 59 were interested, eligible, and randomized [9]. Churches were assigned to an early intervention group (*n* = 39, 66%) or delayed intervention group (*n* = 20, 34%), both involving church member training and program implementation [9]. Of the randomized churches, 92% (54/59) of churches took part in the assessments described next (35/39 intervention and 19/20 delayed). Early and delayed churches did not differ on church-level characteristics, such as church size, predominant race of congregation, and denomination [9]. 

As part of the evaluation activities, two trained research staff visited each church on one day of worship between June and October 2016 (8–12 months after training of early intervention churches but before training of delayed churches) to complete audits of the church physical environment and administer surveys to church attendees. The presence and condition of church facilities related to PA and HE were evaluated by trained research assistants with minimal assistance from church personnel (e.g., locating equipment, unlocking doors) using a recently-developed tool [24]. These church physical environment audits took an average of 19 min to complete [24]. 

For survey data collection, church attendees were informed about the purpose of the study and exclusion criteria and asked to complete an anonymous survey at the conclusion of the church service. Specifically, they received a one-page information sheet with their survey that detailed key points related to consent (e.g., voluntary nature, anonymous survey, numbers to contact the principal investigators and Office of Research Compliance) and were encouraged to keep it and, if they agreed to participate, were asked to proceed with the survey. All attendees were eligible to participate if they were 18 years or older and attended church at least once per month [9]. The surveys were paper-based and required approximately 15 min to complete [9]. If desired, research staff assisted attendees with completion of the survey. Surveys were completed by 1423 attendees; 430 were excluded for a missing covariate, main exposure, or main outcome variable, leaving a sample of 993 attendees [9]. The research protocol was reviewed by the University of South Carolina Institutional Review Board and was granted exempt status in accordance with 45 CFR 46.101(b)(2). All study amendments were submitted to and reviewed by the board, and none were deemed to change the study status.

### 2.2. Measures

The PA and HE physical environment of the church was assessed objectively using the Church Environment Audit Tool, which was developed using a multi-stage process that included a search of existing literature, assessment of related environmental audit tools, expert review, and community advisory board review that resulted in a 6-page tool assessing 7 domains of the physical environment of faith-based organizations [24]. This tool demonstrated strong inter-rater reliability in the current study and can be publicly accessed online: http://prevention.sph.sc.edu/Resources/church-health-environmental-audit-tool.htm [24]. Specifically, it is highly reliable for a broad range of questions across 5 out of the 7 church environment domains: indoor opportunities for physical activity, food preparation equipment, type of kitchen, media assessment, and outdoor opportunities for physical activity [24]. A scoring protocol was developed [25], and used to measure indoor PA opportunities (14 items total), outdoor PA opportunities (9 items total), and HE opportunities (15 items total). Indoor physical activity opportunities refer to presence of fellowship halls/rooms, free weights, rubber bands for stretching, yoga mats, stationary exercise machines, activity/aerobic equipment, active gaming equipment, exercise videos, TV/DVD player, stereo/sound system, sports sets/equipment, bicycles/tricycles/rollerskates/scooters/skateboards, stairs, and PA promotion signage. Outdoor physical activity opportunities refer to running tracks, outdoor lighting, bike racks, community gardens, playgrounds, green spaces, sports fields, sports courts, or vacant land. Finally, healthy eating opportunities (indoor & outdoor) refer to refrigerators, freezers, ovens, stovetops, sinks, dishwashers, microwaves, serving stations, indoor grills, outdoor grills, counter space, fryers (reverse scored), cookbooks, salt shakers (reverse scored), and community gardens. 

To calculate scores for PA and HE opportunities, a value of 1 was assigned to items present in the church. If the item was present, it received additional scores for the following two questions: “Is it usable?” (everything necessary for use is present and nothing prevents use) and “Is it in good condition?” (looks clean and maintained) [25]. Positive ratings for usability and condition were assigned a score of 0.5 and negative ratings assigned a score of -0.5. In total, this study used summary scores for total PA opportunities (max score = 46), including indoor and outdoor PA opportunities, and HE opportunities (max score = 26). 

The church attendee survey collected information related to respondents’ sociodemographic characteristics, perceptions of the PA and HE environment of the church, self-efficacy for PA and HE, and self-reported PA and fruit/vegetable intake according to guidelines [9]. Key sociodemographic characteristics included age group (18–34 years, 35–64 years, & 65+ years), gender (male or female) and education (collapsed into college-educated or not). In addition, the survey collected information on frequency and duration of church participation. Questions included, “How long have you been attending this church?” and “How many times per month do you attend worship services at this church?” For our analyses, frequency of church attendance was converted to weekly or not. 

Self-reported perceptions of the church environment were assessed with multiple items related to whether PA and HE opportunities, messages, or pastor/leader support were perceived to be available as part of church activities or communications [9]. The nine items related to perceptions of PA supports within the church (e.g., “How often were physical activity programs offered at your church?”, “How often were opportunities to be physically active incorporated before, during, or after worship service?”, “How often were opportunities to be physically active incorporated into existing meetings and events?”) demonstrated adequate internal consistency, with a Cronbach’s alpha of 0.93 [26]. Likewise, the six items related to perceptions of fruit/vegetable supports within the church (e.g., “How often were vegetables or vegetable dishes made available to church attendees at church functions that included food?”, “When church bulletins or bulletin inserts or handouts were given to church members, how often were messages about healthy eating provided?”, “How often did the Pastor include messages about healthy eating during church services?”) also demonstrated adequate reliability, with a Cronbach’s alpha of 0.83 [26]. Response options for all items included 1 (rarely or never), 2 (every few months), 3 (about monthly), or 4 (about weekly) and were averaged to create a score for PA perceptions and HE perceptions. 

Self-efficacy for overcoming barriers to PA was measured using five items (e.g., “How confident, or sure, are you that you could participate in regular PA when you feel you don’t have the time?”), which showed adequate internal consistency, with Cronbach’s alpha of 0.88 [26]. Self-efficacy for fruit/vegetable intake in various situations was measured using eight items (e.g., “How confident, or sure, are you that you could eat a healthy snack, like fruits or vegetables, when you’re really hungry?”) had adequate internal consistency, with Cronbach’s alpha of 0.93 [26]. All responses were provided on a 7-point Likert scale (1 = not at all confident, 7 = very confident) and averages were computed for PA and HE self-efficacy, separately.

For self-reported PA, six items about moderate and vigorous PA participation were used from the 2009 Behavioral Risk Factor Surveillance System PA module [27]. This measure was adopted for its sensitivity to differences according to age group [28], and validity to determine whether participants met PA guidelines, defined as greater than 150 min per week of moderate PA or greater than 75 min per week of vigorous PA [29]. For self-reported HE, the questionnaire asked: “About how many cups of fruit (including 100% pure fruit juice) do you eat or drink each day?”, with a parallel question for vegetables [29]. Respondents were given examples of 1-cup equivalents to ensure accurate reporting. This is a conventional measure for fruit/vegetable intake and is sensitive to behavior changes in past faith-based research [30]. Fruit/vegetable intake was grouped as <5 cups per day and >5 cups per day, according to national recommendations and to match past research for this population [9,29].

### 2.3. Analyses

For the first research objective, multiple linear regression examined the association between the church PA physical environment and attendee perceptions of PA supports within the church environment, as well as the church HE physical environment and attendee perceptions of the HE supports within the church environment. For the second research objective, we likewise used multiple linear regression to analyze the relationship between the physical church PA and HE environments and participants’ self-efficacy for PA and HE, respectively. Finally, we employed multiple logistic regression to examine the relationship between the church physical PA environment and meeting PA guidelines, and between the church physical HE environment and meeting recommendations for fruit/vegetable consumption. For linear models, linearity, homoscedasticity, independence, and normality assumptions were checked [31]. For logistic models, binary outcome, linearity between the logit of the outcome and predictors, influential values, and multicollinearity assumptions were checked [31]. For all analyses, we tested whether a multi-level component was necessary to control for non-independence within the church environment using a random effect for church ID. Variance components were estimated for church-level random effects, and interclass correlation coefficients were used to determine whether the random intercept was appropriate. For significant relationships identified in the four aims, we tested for an interaction between weekly church attendance and church environment and presented stratified results for any significant terms. Additionally, all models were adjusted for intervention group, predominant race of the congregation, and attendee age, gender, education, and weekly church attendance. We adjusted for variables that have are related to the outcomes in past research or those that may impact attendee exposure to the church environment. SAS 9.4 software Version 9.4 for Windows (SAS Institute, Cary, NC, USA) was used for all analyses and significance was set at *p* < 0.05.

## 3. Results

Approximately 92% of participating churches had predominantly African American congregations, based on pastor reports. Table 1 reports the mean number (and range) of indoor PA, outdoor PA, and HE opportunities within each church. On average, church environment audits showed that churches had 4.04 indoor PA opportunities, 2.41 outdoor PA opportunities, and 6.67 HE opportunities. Table 2 reports key participant characteristics. Most participants reported being between 35 and 65 years of age (61.4%), female (69.9%), and having at least some college education (55.1%). A majority (77.0%) reported attending church on at least a weekly basis. For church environment, participants’ churches had an average audit score of 13.3 (SD = 5.4) for PA and 14.4 (SD = 2.3) for HE. Church environment scores for PA ranged from 6 to 32 (out of a total score of 46), and for HE ranged from 5 to 19 (out of a total score of 26). Most participants self-reported meeting PA guidelines (71.7%), but relatively few reported meeting recommendations for fruit and vegetable intake (25.9%). With regards to perceptions of the church environment, the mean for PA was 2.3 (SD = 0.9) and the mean for HE was 2.8 (SD = 0.7) out of a maximum score of 4. Participants reported a mean value of 3.7 (SD = 1.5) for self-efficacy for overcoming barriers to PA and 4.8 (SD = 1.4) for self-efficacy for HE out of 7.

**Figure 1:**
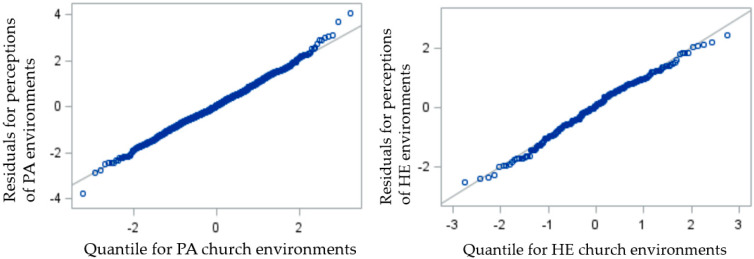
Residual scatter plots for the associations of Church Environment with perceptions of physical activity and healthy eating supports within the church, *N* = 993. Physical activity (PA); healthy eating (HE).

Table 3 displays adjusted associations between the physical church environment related to PA and HE and the three main study outcomes. For the first objective, multi-level analyses were used to control for non-independence within individual churches, since 53.2% (interclass correlation coefficient (ICC) = 0.53) of the variance in the perceptions of PA supports and 48.9% (interclass correlation coefficient (ICC) = 0.49) of the variance in the perceptions of HE supports were explained by church level effects. Results showed that physical church PA and HE environments were associated with higher scores on perceptions of the church environment for PA (B = 0.03, 95% CI = 0.01, 0.05) and HE (B = 0.06, 95% CI = 0.02, 0.09), respectively. For every one unit increase in church environment audit score, perceptions of church environment for PA increased by 0.03 and perceptions of church environment for HE increased by 0.06. Figure 1 provides residual scatter plots for these significant relationships. For the second objective, multi-level analyses were not used to control for non-independence within individual churches, since only 0.7% (ICC = 0.01) of the variance in self-efficacy for PA and 2.4% (ICC = 0.24) of the variance in self-efficacy for HE were explained by church-level effects. Results showed no significant associations between the physical church environment and self-efficacy for PA (B = 0.01, 95% CI = −0.01, 0.03) or HE (B = 0.00, 95% CI = −0.05, 0.05). Finally, multi-level analyses were not used to control for non-independence within individual churches for the third objective, since only 3.1% (ICC = 0.03) of the variance in meeting PA guidelines and 1.0% (ICC = 0.01) of the variance in meeting fruit and vegetable guidelines were explained by church-level effects. Results showed participants with higher physical church environment scores were not significantly more likely to meet PA guidelines (odds ratio (OR) = 1.00, 95% CI = 0.98, 1.03) or fruit and vegetable guidelines (OR = 1.02, 95% CI = 0.96, 1.09).

Table 4 presents the results for research question 4, which assessed the interaction between weekly church attendance and PA and HE opportunities within the church environment for all research outcomes (i.e., perceptions of church environment support, self-efficacy for health behaviors, and self-reported health behaviors). Results showed a significant interaction between weekly church attendance and HE supports within the church but no significant interaction between weekly church attendance and PA supports within the church. For the significant interaction for HE, we conducted stratified analyses: the relationship between HE opportunities and perceptions of HE supports within the church showed a significant relationship among both those who reported weekly church attendance (B = 0.04, 95% CI: 0.01, 0.07) and those who did not (B = 0.10, 95% CI: 0.04, 0.16). Notably, there is a stronger relationship between the healthy eating opportunities within the church and perceptions of healthy eating supports in the church for those who report attending church less frequently than weekly.

## 4. Discussion

This study used multi-level regression models to examine associations between the physical church environment and multiple key health-related constructs. We examined these associations in a church attendee sample that was highly educated and mostly female compared to county level estimates. Our first objective was to examine whether the objectively assessed church environment was related to church attendee perceptions of PA and HE supports within the church environment. We found that greater church PA and HE environment scores were associated with higher perceptions scores for PA and HE, a significant finding for multiple reasons. From a conceptual standpoint, past research studying the relationship between environmental features and perceptions has often reported substantial inconsistency between objective and subjective ratings [33,34]. For example, one study showed residents’ lack of awareness of parks in their neighborhood [33], while other research has observed over-reporting of the availability of food stores [34]. However, similar studies about church settings are lacking and the ‘match’ witnessed in this context is noteworthy [33]. Practically, documenting this association between objective church environment scores and attendees’ perceptions of the PA and HE environment is valuable for understanding the extent to which available facilities and features are perceived by members as accessible, an important precursor to planning effective interventions within this setting.

For our second study objective, we found that the church environments for PA and HE were not significantly related to self-efficacy scores for PA or HE, respectively. Past research has shown that self-efficacy may interact with and be influenced by the environmental resources to support health behaviors [12], but this relationship has yet to be established in the church setting. The self-efficacy scales employed are ostensibly reliable and valid measures for the construct [35]. One explanation for the non-significant findings may be the population under study. Past research has found that those identified as female and African American or black are more likely to have lower self-efficacy scores for exercise compared to males and Whites, respectively [36]. Since our sample of church attendees was predominantly female and most congregations were categorized as majority African American or black, self-efficacy scores may be lower and have less variability. This lack of variability may make it more difficult to establish a significant, positive relationship between church environment and self-efficacy in this population. It is also possible that the physical environmental features alone are not adequate to influence self-efficacy in this setting. The audit tool was broad and included features of the church environment that have the potential to influence PA and HE, but without programming or prompts, the presence of features alone may not be adequate to influence these beliefs and behaviors. Additional features that may impact human comfort and health may also play a role in this relationship, such as indoor air quality [37,38], thermal comfort [39], noise levels [40], and lighting [41], that were not collected as a part of this study. Future research may measure these influences and account for them in subsequent analyses. 

The third objective of this study was to examine whether the church environment was positively associated with church attendee self-reported PA and fruit/vegetable consumption. These self-reports were used to calculate meeting PA and fruit/vegetable intake recommendations, and the population under study reported similar proportions of those who met recommendations compared to levels nationwide [42]. We found that the church environment was not significantly associated with reported PA and fruit/vegetable intake among congregants. Past research in other settings has documented a relationship between environmental factors in workplaces [43], and neighborhoods [44,45], and the PA or HE behaviors of participants. Often, only studies looking at individual environmental features instead of a composite score [43,45], or those who observed participants over time [44], found a significant relationship between environment and behavioral outcomes. In the context of faith-based organizations, more research is needed to determine if relationships exist between church environment characteristics and self-reported behaviors [8,15,46]. 

The final study objective was to observe the interaction of weekly church attendance and church environment, for study outcomes of interest. We found a significant interaction between weekly church attendance and the HE church environment on perceptions of healthy eating supports within the church. Stratified results showed that those who attended church weekly and those that did not both showed a significant relationship between the HE church environment and perceptions of healthy eating supports within the church. Our findings highlight the fact that less frequent church attendance may result in more positive perceptions of HE environments present there. It is possible that regular church users are aware of limitations of HE supports within the church (such as lack of use or use for serving unhealthy items) or are desensitized to supports and messages due to frequent exposure [47]. 

Overall, our study adds to the growing field of research on the relationship between built environment and PA and HE, specifically contributing to knowledge about the impact of environmental attributes on individual-level perceptions in faith-based settings. Past research has shown a mismatch between environmental factors and perceptions in terms of safety and distance to parks [12,33], but we found congruence between church environments and member perceptions. It is important to understand the extent to which available facilities and attributes are known and perceived by members as accessible to promote health and plan interventions within this setting. 

This study also had limitations that merit attention in future research. First, we did not assess certain variables in the work, school, home or other environments that might have been important predictors of health behaviors. Future research should control for other environmental influences to study the unbiased relationships. This study also did not measure participation and engagement in church-related events, particularly those that involve food or PA, or any participation in other non-church interventions to improve PA and HE. Furthermore, the objective church environment audit tool provides a broad summary score of environmental features, and it is possible that only certain elements within the church may influence PA and HE behavior. Future research should examine the impact of specific, church environment features to see which are most impactful on behavioral outcomes. For this study, we also did not account for religiosity when analyzing these results to quantify its impact on attendee responses but did account for the regularity and duration of church attendance which may serve as a proxy measure. Another limitation was the use of self-reported measures of PA and fruit/vegetable consumption, which may be susceptible to reporting biases. For this reason, the results for our latter study objective may under or overestimate the impact of environment on behavior. Lastly, the cross-sectional study design limits conclusions about any causal or temporal relationships; it would be valuable to understand how changes in the church environment impact church member behaviors over time. 

## 5. Conclusions

This innovative study adds to research on the importance of working with faith-based organizations to promote health and, specifically, the impact of church environments on church attendee perceptions. Although we did not find a significant association between the church environment and self-efficacy or self-reported PA and HE, future research may use similar tools within a more diverse sample or examine specific environment factors (versus overall scores) to further investigate these relationships. Likewise, since the sample was fairly homogeneous and the data were cross-sectional, future research should study diverse individuals and environments longitudinally, potentially collecting data on both church environments and the behaviors of people who worship there using objective measures where feasible. Overall, this study concentrated on the role of the church environment since church communities have a strong influence on the health of their attendees [9,10]. Focusing on a rural county in South Carolina allowed us to better understand the importance of the physical church environment on individual-level measures among underrepresented groups, such as rural and African American or black residents. Findings from this study and similar studies can inform future research and public health initiatives in faith-based organizations directed at improving health behaviors and reducing health disparities. 

## Figures and Tables

**Table 1:** Summary of church characteristics, *n* = 54.

Church Characteristic	Mean (SD)	Range
Total physical activity opportunities	6.44 (2.57)	3.00–16.00
Usable	6.02 (2.67)	2.00–16.00
Good condition	6.04 (2.64)	2.00–16.00
Indoor physical activity opportunities ^1^	4.04 (1.95)	1.00–12.00
Usable	3.85 (2.01)	1.00–12.00
Good condition	3.93 (1.98)	1.00–12.00
Outdoor physical activity opportunities ^2^	2.41 (1.32)	0.00–8.00
Usable	2.17 (1.40)	0.00–8.00
Good condition	2.11 (1.37)	0.00–8.00
Healthy eating opportunities ^3^	6.67 (1.18)	2.00–9.00
Usable	6.35 (1.82)	0.00–9.00
Good condition	6.39 (1.80)	0.00–9.00

^1^ Indoor physical activity opportunities refer to presence of fellowship halls/rooms, free weights, rubber bands for stretching, yoga mats, stationary exercise machines, activity/aerobic equipment, active gaming equipment, exercise videos, TV/DVD player, stereo/sound system, sports sets/equipment, bicycles/tricycles/roller skates/scooters/skateboards, stairs, and physical activity (PA) promotion signage. ^2^ Outdoor physical activity opportunities refer to running tracks, outdoor lighting, bike racks, community gardens, playgrounds, green space, sports fields, sports court, or vacant land. ^3^ Healthy eating opportunities (indoor & outdoor) refer to a refrigerators, freezers, ovens, stovetops, sinks, dishwashers, microwaves, serving stations, indoor grills, outdoor grills, counter space, fryers (reverse scored), cook books, salt shakers (reverse scored), & community gardens.

**Table 2:** Sample characteristics of church attendee participants, *n* = 993.

Sample Characteristic	*n*	%
Total	993	100
Age		
18–34 years	131	13.2
35–65 years	610	61.4
>65 years	252	25.4
Gender		
Male	299	30.1
Female	694	69.9
Any college education		
Yes	547	55.1
No	446	44.9
Weekly church attendance		
Yes	765	77.0
No	228	23.0
Meets physical activity guidelines		
Yes	712	71.7
No	281	28.3
Meets fruit/vegetable guidelines		
Yes	257	25.9
No	736	74.1
Randomization		
Delayed intervention	379	38.2
Early intervention	614	61.8
Duration of church attendance, years (Mean, SD)	32.7	21.6
Church physical activity environment score (Mean, SD)	13.3	5.4
Church healthy eating environment score (Mean, SD)	14.4	2.3
Perceptions of church physical activity environment (Mean, SD) ^1^	2.3	0.9
Perceptions of church healthy eating environment (Mean, SD) ^1^	2.8	0.7
Self-efficacy for physical activity (Mean, SD) ^2^	3.7	1.5
Self-efficacy for healthy eating (Mean, SD) ^2^	4.8	1.4

^1^ Perceptions of physical activity and healthy eating supports within the church environment scores ranged from 1 to 4. ^2^ Self-efficacy for physical activity and healthy eating scores ranged from 1 to 7.

**Table 3:** Associations of church environment with physical activity and healthy eating environment perceptions, self-efficacy, and self-reported behavior, *n* = 993.

Outcome Variables	Bivariate Associations ^1^ t/r (*p*-Value)	B or OR (SE)	95% CI for B/OR	R^2^ Adjusted (R^2^ Crude) ^2^
Perceptions ^3^				
Physical activity supports	0.31 (*p* < 0.01) *	0.03 (0.01)	0.01, 0.05 *	0.19 (0.01) ^4^
Healthy eating supports	0.22 (*p* < 0.01) *	0.05 (0.02)	0.02, 0.09 *	0.22 (0.00) ^4^
Self-efficacy ^5^				
Physical activity	0.07 (*p* = 0.02) *	0.02 (0.02)	−0.02, 0.06	0.05 (0.00)
Healthy eating	0.00 (*p* = 0.94)	−0.01 (0.02)	−0.05, 0.03	0.05 (0.00)
Meets guidelines ^6^				
Physical activity ^7^	0.26 (*p* = 0.79)	1.00 (0.01)	0.97, 1.03	0.02 (0.00)
Healthy eating ^8^	−0.72 (*p* = 0.47)	1.02 (0.03)	0.95, 1.09	0.03 (0.00)

Odds Ratio (OR); * *p* < 0.05; ^1^ T-tests were used for categorical variables (i.e., meetings physical activity (PA) and healthy eating (HE) guidelines) and Pearson’s correlations for continuous variables (i.e., self-efficacy for PA and HE, and perceptions of PA and HE supports within the church) to assess their relationship with church PA and HE environment. ^2^ R^2^ values pertain to models where all adjustment variables were included, as well as crude models where only the church environment variables were included.^3^ Multi-level linear regression, adjusting for church clustering, randomization, predominant race of church members, age, gender, college education, and weekly church attendance. ^4^ R^2^ values for multi-level models with random effects are based on a method developed by Magee (1990) [32] using −2 log likelihood estimates of the full and intercept only models. ^5^ Linear regression, adjusting for randomization, predominant race of church members, age, gender, college education, and weekly church attendance. ^6^ Logistic regression, adjusting for randomization, predominant race of church members, age, gender, college education, and weekly church attendance. ^7^ PA guidelines defined as greater than 150 min per week of moderate PA or greater than 75 min per week of vigorous PA. ^8^ Fruit and vegetable intake guidelines defined as 5 or more cups/day.

**Table 4:** Interaction between objective church environment and weekly church attendance for perceptions of church environment supports, self-efficacy, and health behaviors, *n* = 993.

Variables	B or Odds Ratio (SE)	95% CI	R^2^
Interaction Term Estimates—Perceptions of Church Environment Supports as Outcome ^1,2^			
Weekly Church Attendance × Physical Activity Opportunities	0.00 (0.01)	(0.00, 0.01)	0.19 ^3^
Weekly Church Attendance × Healthy Eating Opportunities	−0.04 (0.02)	(−0.08, −0.01) *	0.23 ^3^
Interaction Term Estimates—Self-Efficacy for Health Behaviors as Outcome ^1,4^			
Weekly Church Attendance × Physical Activity Opportunities	−0.03 (0.02)	(−0.06, 0.02)	0.06
Weekly Church Attendance × Healthy Eating Opportunities	−0.04 (0.05)	(−0.14, 0.06)	0.05
Interaction Term Estimates—Health Behaviors as Outcome ^1,5^			
Weekly Church Attendance × Physical Activity Opportunities	0.00 (0.02)	(−0.02, 0.01)	0.02
Weekly Church Attendance × Healthy Eating Opportunities	0.03 (0.05)	(−0.07, 0.12)	0.03

* *p* < 0.05; ^1^ Referent category for weekly church attendance is not attending weekly. Estimates represent the effect of weekly church attendance. ^2^ Multi-level linear regression, adjusting for church clustering, randomization, predominant race of church members, age, gender, college education, and age by objective, physical activity environment. ^3^ R2 values for multi-level models with random effects are based on a method developed by Magee (1990) [32] using −2 log likelihood estimates of the full and intercept only models. ^4^ Linear regression, adjusting for randomization, predominant race of church members, age, gender, college education, and weekly church attendance. ^5^ Logistic regression, adjusting for randomization, predominant race of church members, age, gender, college education, and weekly church attendance.
